# The Therapeutic Potential of Pulsed Electromagnetic Fields (PEMF) and Low-Intensity Pulsed Ultrasound (LIPUS) in Peripheral Nerve Regeneration: A Comprehensive Review

**DOI:** 10.3390/ijms26199311

**Published:** 2025-09-24

**Authors:** Danuta Piotrzkowska, Mateusz Siwak, Julia Adamkiewicz, Lukasz Dziki, Ireneusz Majsterek

**Affiliations:** 1 Department of Clinical Chemistry and Biochemistry, Medical University of Lodz, 90-419 Lodz, Poland; mateusz.siwak@umed.lodz.pl (M.S.); ireneusz.majsterek@umed.lodz.pl (I.M.); 2 Department of General and Oncological Surgery, Medical University of Lodz, Pomorska 251, 92-213 Lodz, Poland; julia.adamkiewicz@umed.lodz.pl (J.A.); lukasz.dziki@umed.lodz.pl (L.D.)

**Keywords:** pulsed electromagnetic field (PEMF), low-intensity pulsed ultrasound (LIPUS), peripheral nerve regeneration, nerve injury, Schwann cells, neuropathy

## Abstract

Peripheral nerve injuries (PNIs) present significant clinical challenges, often leading to severe motor, sensory, and autonomic dysfunction, with profound impacts on patient quality of life and considerable healthcare costs. This review synthesizes current knowledge on the therapeutic potential of Pulsed Electromagnetic Fields (PEMF) and Low-Intensity Pulsed Ultrasound (LIPUS) as non-invasive modalities for promoting peripheral nerve regeneration. We explore their cellular and molecular mechanisms of action, discuss optimal treatment parameters, and present evidence from preclinical and clinical studies, including their synergistic effects with other therapies and emerging applications beyond neurological repair. Clinical studies have shown that PEMF therapy can significantly reduce neuropathic pain and improve functions, whereas LIPUS demonstrates the ability to enhance nerve conduction.

## 1. Introduction

Peripheral nerves play a key role in the body, connecting the brain and spinal cord to muscles and organs. Their damage can lead to severe motor, sensory and autonomic disorders. Damage to peripheral nerves can result from diseases of the nervous system or systemic conditions (e.g., diabetes), as well as from complications due to poisoning (e.g., chemotherapy-induced peripheral neuropathy) or infections. Mechanical stimuli, such as pressure or trauma, can also cause neuropathy. Mechanical injuries to peripheral nerves most often affect superficial or anatomically poorly protected nerves, mainly in the upper limbs. Typical mechanical injuries to peripheral nerves include bone dislocations and fractures, cuts and lacerations, gunshot injuries and electrical trauma [1,2].

Furthermore, peripheral nerve damage associated with chronic diseases is commonly seen in diabetes, often leading to the condition known as diabetic foot. This results from damage to sensory fibers (which increases the risk of injury), autonomic fibers (which impairs tissue nutrition), and the effects of chronic hyperglycaemia (which exacerbates nerve damage and hinders wound healing). Other diseases that can cause peripheral nerve damage include Guillain–Barré disease, multiple sclerosis, and poliomyelitis. Symptoms of peripheral nerve damage depend on the type of nerve fibers damaged and the site of injury. They may include pain (sharp or burning), tingling, numbness, muscle weakness, muscle atrophy, sensory disturbances (e.g., touch, pain, temperature) and autonomic disturbances (e.g., excessive sweating, changes in skin temperature) [1,2].

The consequences of peripheral nerve damage can be severe and include permanent disability, loss of motor function, loss of sensation, chronic pain, depression and anxiety, social isolation, and loss of ability to work. The absence of effective treatment frequently results in long-term disability and places a substantial financial burden on healthcare systems [3,4].

Peripheral nerve injuries (PNI) are a widespread clinical problem with significant systemic impact. Their global incidence is estimated at 13–23 cases per 100,000 population annually, emphasizing their scale [5]. In 2023, the European PNI treatment market alone reached a value of $443 million, with projections for further growth, indicating a rising economic burden [3]. Furthermore, recent 2024 data show that 0.8% of patients following joint arthroplasty procedures have PNI complications, underscoring the clinical challenges associated with these injuries [4].

Although the peripheral nervous system possesses a spontaneous regenerative capacity, axonal regrowth is typically slow—progressing at a rate of approximately 1–3 mm per day and often incomplete. This regenerative process is frequently impeded by fibrosis and scar tissue formation, which act as physical and biochemical barriers to axonal extension and reinnervation of target structures [5,6].

Schwann cells are central to nerve regeneration, as they reprogram into a repair phenotype after injury, clearing myelin debris, secreting neurotrophin, and forming guiding structures (Bands of Büngner) that direct axonal regrowth [7]. The microenvironment, shaped by Schwann cells and extracellular matrix components, provides essential molecular cues and spatial organization for regenerating axons, which is why therapies like PEMF and LIPUS—capable of modulating cellular activity and the local milieu—are promising for enhancing nerve repair [8,9].

Advancements in medical technology have led to various repair strategies, broadly categorized into surgical (e.g., nerve transfer, grafts, conduits) and non-surgical approaches (e.g., magnetic and electric field therapy, He-Ne laser therapy, electrophotoluminescence) [10,11].

In recent years, intensive research has focused on finding more effective and less invasive treatment methods to minimize the negative effects of PNI. The diversity of PNI causes and severity requires a holistic approach that includes both surgical interventions and non-invasive supportive therapies. This demand for innovative solutions is driving the development of physical therapies that can stimulate the body’s natural repair processes. In this review, we will discuss in detail how PEMF and LIPUS fit into this paradigm, offering new treatment perspectives that could change the approach to caring for patients with PNI.

## 2. Therapeutic Modalities: PEMF and LIPUS

Physical therapies, a specialized domain within physiotherapy, employ biophysical modalities—including thermal, cryogenic, electrical, photonic, and electromagnetic agents—to elicit therapeutic effects. These interventions primarily induce analgesic, anti-edematous, and anti-inflammatory responses through both localized and systemic physiological mechanisms. Table 1 provides a detailed comparison of the key parameters and mechanisms of action for PEMF and LIPUS therapy.

### 2.1. Pulsed Electromagnetic Field (PEMF)

Pulsed electromagnetic field (PEMF) therapy is a non-invasive therapy method using alternating electromagnetic fields to stimulate cells and tissues, and it has gained increasing recognition in regenerative medicine [12,13]. Its therapeutic potential includes alleviating inflammation, supporting tissue repair, and accelerating healing [14,15]. Research into its tissue-stimulating potential began in the 1970s [12]. PEMF therapy evolved from early studies on bone fracture healing, distinguishing itself from other magnetic field-based methods by its dynamic, pulsed nature, which allows for precise modulation of cellular responses. Key parameters such as frequency, intensity, and waveform shape (e.g., sinusoidal, trapezoidal) are critical for achieving optimal biological effects.

Unlike other physical stimuli, PEMF offers deep tissue penetration, enabling interactions at cellular, tissue, and organ levels [13,16]. Its efficacy and broad applicability across medical disciplines have been substantiated by extensive global research, establishing its use as a standard for certain conditions [13,16].

The approval of PEMF by the U.S. Food and Drug Administration (FDA) for treating bone non-unions in 1979 solidified its clinical foundation, paving the way for further research into its potential in neuroregeneration.

Clinical studies consistently demonstrate that PEMF exhibits anti-inflammatory and anti-edematous properties by modulating inflammatory processes and enhancing microcirculation through vasodilation and angiogenesis [13,14,15,16]. PEMF also exerts potent analgesic effects for both acute and chronic conditions, reduces muscle spasticity, and stimulates tissue regeneration in soft tissue, connective tissue, cartilage, and bone [13,16].

In orthopedics, PEMF therapy is widely employed for fracture treatment, including fresh fractures and those with delayed fusion [17]. For instance, Faldini et al. demonstrated that PEMF therapy accelerated fracture healing and reduced pain in 94% of patients with femoral neck fractures compared to controls [17]. Furthermore, in the context of osteoarthritis, PEMF can mitigate its progression by activating the Sirt1/NF-κB pathway, leading to reduced inflammation and tissue repair [18]. In mouse models, PEMF treatment attenuated osteoarthritis progression by inhibiting TNF-α and IL-6 signaling, thereby alleviating pain, decreasing cartilage degeneration, and improving bone microarchitecture [19]. Meta-analyses confirm that PEMF therapy positively influences pain, stiffness, and physical function in osteoarthritis patients, with more pronounced short-term effects [20]. Overall, PEMF promotes bone healing and joint preservation by augmenting the structural integrity of the bone and cartilage extracellular matrix, reducing inflammation, and enhancing repair, partly through adenosine A2A and A3 cell membrane receptor signaling [21]. Optimal parameters for effective PEMF therapy typically involve trapezoidal or sawtooth signals with peak magnetic field intensities of 1.2 mT to 2 mT and repetition frequencies of 15 Hz to 75 Hz [22]. Although PEMF demonstrates potential benefits for musculoskeletal disorders, additional research is required to establish standardized treatment protocols.

PEMF therapy is also being studied in other areas of medicine beyond musculoskeletal uses. It has shown promise in aiding hematoma resolution and alleviating inflammation following intracerebral hemorrhage, potentially improving neurological function. PEMF can also reduce interleukin-6 expression in intervertebral disk cells via NF-κβ and p38 MAPK pathways, benefiting intervertebral disk degeneration treatment [23]. In oncology, PEMF may inhibit cervical cancer progression by decreasing IL-37 expression and improving CD8+ cell function and can enhance the antiproliferative effect of doxorubicin on breast cancer cells by increasing cell cycle arrest and DNA damage [24]. Additionally, PEMF may reduce septic shock by downregulating pro-inflammatory cytokine gene expression [25]. In cardiology and angiogenesis, PEMF stimulates FGF-2 release by endothelial cells, offering potential applications in ischemic therapies [26]. In neurology, PEMF effectively reduces microglial neuroinflammatory processes through JNK MAPK pathway activation [27].

### 2.2. Low-Intensity Pulsed Ultrasound (LIPUS)

Low-Intensity Pulsed Ultrasound (LIPUS) therapy is another non-invasive modality employing low-intensity ultrasound waves to stimulate cells and tissues. It is characterized by good tissue penetration and a low risk of side effects. It is used for conditions like those treated by PEMF, including fractures, bone, joint, and tendon diseases, accelerating tissue regeneration, and reducing pain and inflammation. Furthermore, LIPUS promotes fibroblast differentiation and the repair of cartilage, ligaments, and tendons [28,29,30]. The U.S. Food and Drug Administration (FDA) approved LIPUS in 1994 and 2000 as a safe and non-invasive treatment for fresh fracture healing and non-unions [31].

The mechanism of LIPUS, while not fully understood, is believed to primarily involve non-thermal phenomena such as microbubble and microjet formation induced by cavitation, acoustic streaming, and mechanical stimulation [32].

Unlike diagnostic ultrasound, which uses high intensity for imaging, LIPUS is specifically designed to stimulate cells through mechanotransduction—the process by which cells convert mechanical stimuli into biochemical signals. Key to this process are phenomena like cavitation (the formation and collapse of microbubbles), acoustic streaming, and micro-fluidic flows, all of which influence cell membrane integrity, increase ion channel permeability, and stimulate neurotrophin secretion.

At the cellular level, LIPUS activates signaling pathways critical for cell proliferation, differentiation, and migration. It also modulates the inflammatory response by influencing cytokine expression and inhibiting NF-κB and MAPK pathways [33,34]. LIPUS additionally stimulates the secretion of extracellular vesicles (EVs) and microRNAs, which can regulate immune cell functions and repair processes [35,36].

In clinical practice, LIPUS has been shown to accelerate fracture healing, promote soft tissue regeneration, improve angiogenesis, support diabetic wound healing, and reduce inflammation and pain [33,36,37,38]. Beyond the musculoskeletal system, LIPUS exhibits beneficial effects on kidney, heart, and adipose tissue function. It also holds promise in neurological disease therapy, where it supports neurogenesis and repair of damaged structures. Moreover, LIPUS can enhance the efficacy of cellular therapies by improving exosome absorption and stimulating angiogenesis [36,39]. Due to its broad spectrum of action and high safety profile, LIPUS is increasingly used as an adjunct to traditional treatment methods in various medical therapies [33,34,38].

## 3. Mechanisms of Action at the Cellular and Molecular Level

### 3.1. PEMF Mechanisms

The precise mechanisms of pulsed electromagnetic field (PEMF) action are still being elucidated, but unlike single-pathway drug therapies, PEMF appears to induce complex, cascading intracellular responses. Each electromagnetic pulse mobilizes ion flow within the cell membrane, leading to its depolarization, repolarization, and hyperpolarization. This transiently opens ion channels for calcium (Ca^2+^) and sodium (Na^+^), initiating a cascade of biochemical pathways in the cytoplasm [40,41]. The increase in cytoplasmic concentration activates enzymes, such as protein kinases (e.g., protein kinase C) and phosphatases, including calcineurin [42]. These enzymes transmit the signal to the cell nucleus, where they activate transcription factors through phosphorylation or dephosphorylation. Simultaneously, the activation of enzymes leads to the production of second messengers, such as cAMP and cGMP, which amplify the signal and activate subsequent signaling pathways, including MAPK and SMAD. Consequently, this leads to the transcription of genes responsible for cell differentiation and proliferation, such as RUNX2, DLX5, and BMP2 [43,44]. The increased expression of these genes results in the production of proteins and growth factors that support regenerative processes, including osteogenesis and extracellular matrix mineralization, while restoring homeostasis [40].

**Figure 1:**
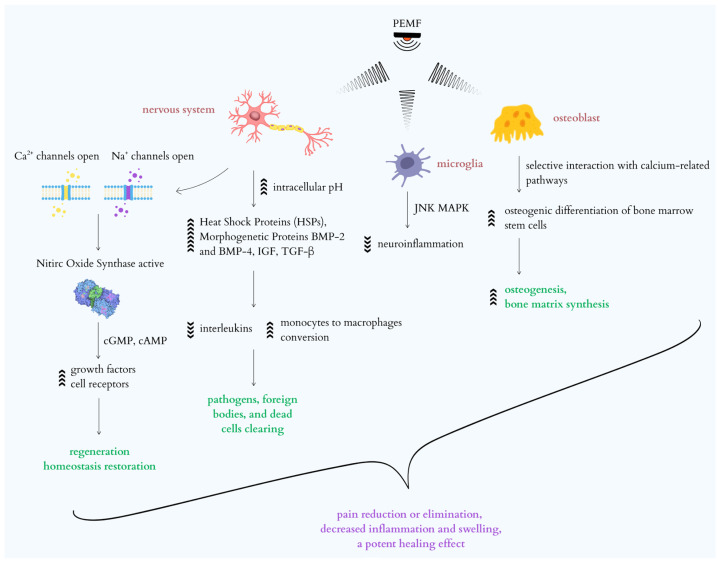
Cellular and molecular mechanisms of Pulsed Electromagnetic Fields (PEMF) in peripheral nerve regeneration and inflammation reduction.

PEMF exposure also leads to an increase in intracellular pH, a decrease in catabolic activity, and a stimulation of anabolic processes. Its effect on cellular processes is linked to gene expression regulation; studies indicate that PEMF can upregulate heat shock proteins (HSPs), which protect cells from stress and death and are vital for proper protein folding [45,46]. PEMF therapy further stimulates the secretion of active factors such as morphogenetic proteins BMP-2 and BMP-4, as well as IGF and TGF-β [14,15,47]. Concurrently, it reduces inflammatory components (interleukins) and enhances the conversion of monocytes into macrophages, thereby clearing pathogens, foreign bodies, and dead cells from diseased sites [46,48]. This multifaceted action allows PEMF to block necrosis and promote multi-level regeneration, contributing to humoral and immune activity throughout the body. Macroscopically, this translates to pain reduction or elimination, decreased inflammation and swelling, and a potent healing effect [46,48]. Low-frequency, low-energy PEMF specifically achieve anti-inflammatory effects by increasing adenosine A2A and A3A receptor expression in various cells and tissues, leading to reduced pain and inflammation [48]. PEMF stimulates osteoblast activity, promoting osteogenesis and bone matrix synthesis through diverse pathways, including selective interaction with calcium-related pathways to favor osteogenic differentiation of bone marrow stem cells [49]. The synergistic effect of PEMF and BMP-2 further enhances osteogenic differentiation of human bone marrow mesenchymal stem cells by modulating BMP signaling components [50]. In the context of neuroinflammation, PEMF shows therapeutic potential, mainly by modulating the JNK MAPK signaling pathway in microglial cells [30]. The JNK MAPK and NF-κB pathways play a central role in regulating the inflammatory response of microglia after nerve injury, and their excessive activation leads to chronic inflammation that inhibits regeneration. PEMF, by inhibiting JNK phosphorylation, limits microglial activation and the production of pro-inflammatory cytokines, such as IL-1β and TNF-α, which results in the reduction in neuroinflammation and promotes nerve regeneration [51,52]. Additionally, inhibiting the JNK pathway can lead to a decrease in NF-κB activity, further restricting the inflammatory response [51,52]. The inhibition of these pathways by either PEMF or LIPUS limits the production of pro-inflammatory cytokines, reduces oxidative stress, and decreases pyroptosis activity, which promotes the repair of nerve tissue and improves motor function. The multifaceted effects of PEMF, including its non-thermal phenomena and its influence on signaling pathways, are illustrated in Figure 1.

### 3.2. LIPUS Mechanisms

The precise mechanism of low-intensity pulsed ultrasound (LIPUS) is multifaceted, encompassing both mechanical and molecular phenomena. LIPUS primarily operates through non-thermal effects such as acoustic streaming, cavitation, and the formation of microbubbles and microjets induced by mechanical stimulation [32]. These physical forces translate into a diverse range of biological effects, including regenerative, angiogenic, and potent anti-inflammatory responses in various tissues, including bone, cartilage, and tendons.

At the cellular level, LIPUS effectively modulates the inflammatory response by directly inhibiting key signaling pathways, specifically NF-κB and MAPK. This inhibition leads to a cascade of molecular events, including a reduction in the phosphorylation of the p65 subunit of NF-κB and MAPK kinases (p38, JNK) [53,54,55]. The consequence is a significant downregulation of genes encoding pro-inflammatory cytokines, such as TNF-α and IL-1β, as well as proteins associated with pyroptosis (e.g., NLRP3, caspase-1, GSDMD) [56]. This targeted restriction of inflammatory mediators is crucial for mitigating neuroinflammation, limiting secondary damage after nerve injury, and creating a more favorable microenvironment for nerve regeneration [53,54,55].

LIPUS also plays a key role in intercellular communication by stimulating the secretion of extracellular vesicles (EVs), including exosomes, from mesenchymal cells. These EVs are enriched with anti-inflammatory microRNAs, such as miR-328-5p and miR-487b-3p (1234). The altered miRNA cargo within the EVs allows them to be taken up by immune cells, such as macrophages and T lymphocytes, which effectively modulates their activity, proliferation, and inflammatory responses [35,36,57,58,59].

Furthermore, LIPUS promotes tissue repair by stimulating the differentiation of fibroblasts, which leads to the effective regeneration of cartilage, ligaments, and tendons [28,30]. In the case of fibroblasts, LIPUS can both inhibit their pathological proliferation (e.g., in the synovial membrane of joints) and promote their migration and repair in tissues such as skin or ligaments, depending on the tissue context [60]. In cartilage regeneration, LIPUS supports the differentiation of mesenchymal cells into chondrocytes by activating signaling pathways such as TNF, Wnt/β-catenin, PI3K/Akt, MAPK/ERK, Notch, and Sonic Hedgehog, which regulate cell proliferation, differentiation, and migration [39,61,62]. Additionally, LIPUS activates pathways related to osteogenesis (e.g., BMP-2/Smad), supporting bone repair and accelerating fracture healing [63,64].

**Figure 2:**
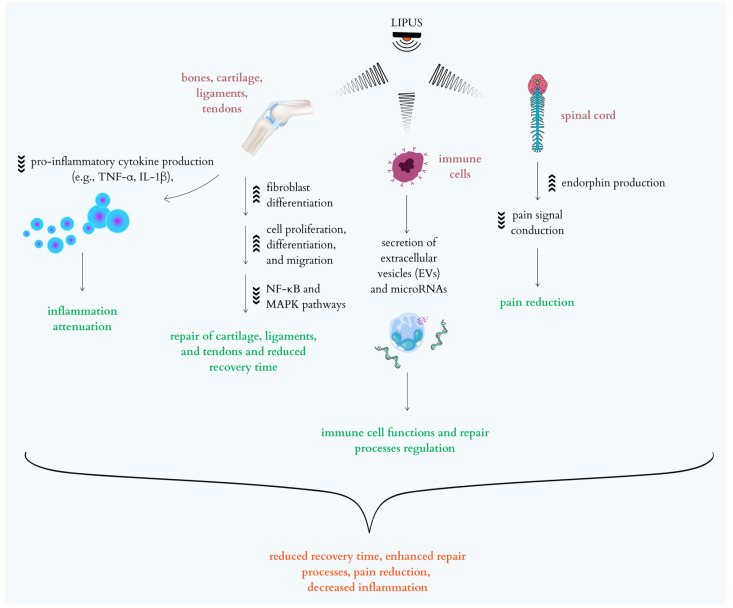
Cellular and molecular mechanisms of Low-Intensity Pulsed Ultrasound (LIPUS) therapy.

Therefore, the comprehensive action of LIPUS includes both the direct stimulation of reparative processes at the cellular and molecular levels, as well as immunomodulation and pain relief, for example, by stimulating endorphin production and improving microcirculation [34,65]. These mechanisms make the action of LIPUS multi-level and significantly more complex than a mere mechanical effect. Ultimately, these processes lead to reduced recovery time and improved function of damaged tissues [34,39,60,62,65]. The multifaceted effects of LIPUS, including its non-thermal phenomena and its influence on signaling pathways, are illustrated in Figure 2.

## 4. Effects on Peripheral Nerve Regeneration

### 4.1. PEMF Effects on Nerve Regeneration

Schwann cells, as peripheral glial cells, are fundamental to the regeneration of damaged peripheral nerves by regulating blood flow and providing a conducive environment for nerve fiber repair [5,66]. PEMF application enhances regenerative processes at the cellular level by increasing ionic permeability of cell membranes and triggering intracellular signaling cascades [67]. One hypothesis suggests PEMF mimics an intracellular calcium wave, initiating the expression of key proteins for axon elongation (RAG) at axon break sites [68]. Through a calcium-dependent mechanism, elevated levels of brain-derived neurotrophic factor (BDNF) and its receptor TrkB upregulate the expression of regeneration-associated genes (RAGs) via activation of the cyclic AMP (cAMP) signaling pathway. This effect is further enhanced by BDNF-mediated inhibition of phosphodiesterases, which normally degrade intracellular cAMP levels [69].

The mechanical and electrical stimuli from PEMF can influence the conformation of ion channels and receptors, leading to their activation. This mechanotransduction process is crucial for converting external physical energy into internal cellular responses. Consequently, this cascade of events modulates gene expression patterns, promoting the synthesis of proteins vital for nerve repair and growth. In the cell body, PEMF elevates BDNF levels, which in turn triggers proteins like Tα1 tubulin and Growth-Associated Protein-43 (GAP-43) [68]. BDNF, produced by motor neurons, DRG neurons, and Schwann cells, is essential for normal development, synaptic plasticity, and learning/memory. Huang’s research showed that PEMF in Schwann cells increases Nerve Growth Factor (NGF) expression, leading to enhanced myelination of damaged axons induced by elevated BDNF levels [70]. This myelination-promoting effect was also observed in the central nervous system in an animal model of multiple sclerosis [71]. The synergistic application of PEMF with other treatments and exercise may yield even better nerve regeneration outcomes [72].

PEMF demonstrates synergistic effects with cellular therapies, such as those involving stem cells or Schwann cells, significantly enhancing reparative outcomes. Studies in animal models have demonstrated that the combination of PEMF therapy with cell transplantation or growth factor administration significantly enhances axonal regeneration and functional recovery compared to either intervention alone. However, the efficacy of PEMF is highly dependent on the precise selection of stimulation parameters; excessively long or intense application may not provide additional benefits and can even induce oxidative stress, potentially delaying regeneration [73,74].

While Pulsed Electromagnetic Field (PEMF) therapy holds significant promise for tissue regeneration and pain management, its clinical application faces considerable limitations. A major challenge is the heterogeneity of therapeutic parameters—including frequency, intensity, and exposure time—which are inconsistently reported across the literature [75,76]. This variability hinders the direct comparison of study outcomes, making it difficult to establish standardized, evidence-based treatment protocols. Consequently, the lack of clarity regarding optimal parameters contributes to the observed discrepancies in reported clinical effects.

Furthermore, the therapeutic efficacy of PEMF is not universally consistent. For example, some preclinical studies on animal models have failed to show a significant positive effect on sciatic nerve regeneration [77]. This suggests that the therapy’s effectiveness may be highly dependent on the specific application and context. The reliance on preclinical data poses a significant translational challenge, as results from animal and cellular models may not directly correlate with human clinical outcomes. These factors, along with potential risks such as localized heating of metal implants and contraindications for patients with pacemakers, necessitate a cautious and well-informed approach to the use of PEMF in clinical practice [76]. Some research suggests that therapeutic effects may be limited to the immediate post-injury period or dependent on individual biological factors and specific stimulation parameters [78,79]. In certain instances, prolonged PEMF exposure did not yield significant improvements in nerve function and was even associated with increased oxidative stress. Therefore, while PEMF represents a promising non-invasive modality for peripheral nerve regeneration, its optimal application requires further rigorous, well-designed clinical studies.

### 4.2. LIPUS Effects on Nerve Regeneration

Schwann cells are pivotal in peripheral nerve regeneration, and LIPUS demonstrates potential in aiding this process. LIPUS increases the secretion of neurotrophins by Schwann cells, promoting axonal regrowth [11,80,81]. Studies confirm that LIPUS application enhances damaged nerve regeneration by altering the expression of genes related to neurotrophins, cytokines, and promyelinating genes during the process [80,81]. Peng’s work showed that LIPUS therapy in Schwann cells can increase the number, diameter, and myelination of axons distal to the injury site, improving Nerve Conduction Velocities (NCV) and Compound Muscle Action Potentials (CMAP) [82]. LIPUS also regulates pro-inflammatory cytokines; it inhibits their expression, thereby attenuating the inflammatory response during nerve regeneration [83,84]. Pro-inflammatory cytokines (e.g., TNFα, IL-6), released by Schwann cells hours after injury, recruit macrophages to clear myelin debris and restructure the extracellular matrix [83,84]. Ito’s study demonstrated that LIPUS therapy inhibited TNFα and IL-6 expression seven days post-nerve injury in rats [80]. Other studies confirm that LIPUS promotes myelin thickening and increases NCV in peripheral nerves after injury [32,85]. Yue’s research indicated that in vitro LIPUS application to Schwann cells increases the expression of key myelination proteins, including ErbB3 (receptor for neuregulin 1), EGR2 (transcriptional regulator of Schwann cell myelination), and Myelin Basic Protein (MBP) [86].

Beyond Schwann cell repair, LIPUS is also suspected to act directly on axons [32,85]. It promotes neurite outgrowth by reducing the expression of axon regeneration inhibitors [32,85]. Recent study showed that LIPUS application to dorsal root ganglion neurons resulted in a twofold increase in neurite outgrowth compared to controls, likely mediated through activation of the Netrin-1/DCC signaling pathway [87]. Furthermore, LIPUS is suggested to promote axon outgrowth by decreasing the expression of semaphorin 3A (an axon regeneration inhibitor) and GSK-3β (a potential inhibitor of axon outgrowth) [86]. Preclinical studies consistently indicate that LIPUS supports peripheral nerve regeneration after injury without negative side effects such as limitation or impairment of regeneration. LIPUS also supports neurogenesis and the repair of damaged neurological structures [80,81].

Extensive preclinical and clinical studies over the past two decades have investigated LIPUS effects on peripheral nerve damage [88,89]. Research on the application of LIPUS therapy in peripheral nerve regeneration is diverse in terms of the type of damage model, the LIPUS parameters, and the evaluation of the therapy used [32]. Despite this heterogeneity, key findings on optimal LIPUS conditions have emerged. Optimal LIPUS intensity for nerve regeneration is typically between 200 and 500 mW/cm^2^; lower intensities (≤100 mW/cm^2^) show no beneficial effect, while higher intensities (≥1 W/cm^2^) can inhibit regeneration. For enhanced nerve regeneration, daily or every-other-day application for 1 to 10 min is recommended [32].

## 5. Limitations, Side Effects, and Contraindications of PEMF and LIPUS in Peripheral Nerve Regeneration

Physical therapies such as pulsed electromagnetic fields (PEMF) and low-intensity pulsed ultrasound (LIPUS) are gaining prominence in regenerative medicine. Nevertheless, their practical application is associated with specific limitations, potential side effects, and contraindications. Although in vitro studies on Schwann cells have not demonstrated cytotoxicity or genotoxicity, suggesting their safety at the cellular level [90], the scientific literature emphasizes the need for caution. One of the main challenges is the lack of sufficient, long-term clinical trials in humans to confirm their efficacy and safety. Additionally, key therapeutic parameters like frequency, intensity, and exposure time are often inconsistent, which complicates the comparison of results and the establishment of standardized treatment protocols [90]. In the context of peripheral nerve regeneration, another limitation is the insufficient translation of preclinical findings to clinical effects in humans, as most data are derived from animal or cellular models [90,91,92]. Moreover, the effectiveness of these therapies may be limited in cases of extensive nerve damage or long-term denervation [93,94]. It is also crucial to be aware of potential side effects and contraindications: PEMF should not be used in patients with electronic implants (such as pacemakers) or in pregnant women, while LIPUS is contraindicated near tumors and during pregnancy [92]. In conclusion, although PEMF and LIPUS offer promising potential, their full and safe clinical implementation requires further well-designed studies to optimize parameters and fully understand their mechanisms of action [90,92]. Table 2 presents a comparison of the key limitations, potential side effects, and contraindications associated with PEMF and LIPUS therapies.

## 6. Conclusions and Future Perspectives

Both PEMF and LIPUS exert their therapeutic effects at the cellular and molecular levels, stimulating regenerative and repair processes [9]. PEMF enhances blood flow, reduces inflammation, and stimulates nerve cell activity, while LIPUS supports the growth and regeneration of nerve tissue [11,80,81].

Furthermore, recent studies show that both PEMF and LIPUS can directly influence the proliferation of Schwann cells and modulate the expression of key genes involved in nerve repair, such as neurotrophins (NGF, BDNF) and anti-inflammatory cytokines, thereby creating a more favorable environment for nerve regeneration. Clinical studies have shown that PEMF therapy can significantly reduce neuropathic pain and improve functional recovery [9].

Modern devices generating PEMF and LIPUS are increasingly adopted in regenerative medicine, yielding promising results in treating peripheral neuropathy stemming from various causes, including metabolic diseases (e.g., diabetes), mechanical injuries, or infections. In diabetic neuropathy, particularly with diabetic foot development, PEMF and LIPUS therapies can be crucial in improving sensation and nerve function, fostering better wound healing, and reducing amputation risk. For mechanical nerve damage (e.g., fractures, gunshot wounds), PEMF and LIPUS can accelerate nerve regeneration and enhance motor and sensory functions. Furthermore, in conditions such as chemotherapy-induced peripheral neuropathy, Guillain–Barré syndrome, multiple sclerosis, and poliomyelitis, both PEMF therapy and LIPUS have shown potential in promoting neural repair and enhancing patient quality of life [1,2]. The cumulative body of evidence highlights the substantial potential of PEMF and LIPUS in peripheral nerve regenerative therapy, positioning them as promising modalities in the evolving landscape of neuropathy treatment. Importantly, the effectiveness of PEMF and LIPUS depends on specific stimulation parameters, highlighting the need for further research to optimize protocols for different types of nerve injuries and patient populations. Their increasing clinical adoption, along with encouraging preclinical and clinical outcomes, underscores the critical need for continued research to elucidate their underlying mechanisms of action and to optimize therapeutic parameters across diverse clinical contexts. Future work should consider developing standard protocols tailored to specific types of nerve injuries. Another promising research direction involves examining the interaction between LIPUS and PEMF and their combined effects on cellular activity and nerve regeneration. Siwak et al. reported the greatest reduction in IL-6 expression—up to 60% in Schwann cells—following combined treatment with PEMF at 1000 Hz and LIPUS at 40 kHz. These findings suggest that the simultaneous application of both fields may enhance their anti-inflammatory effects [90]. Future studies should focus on optimizing stimulation parameters, clarifying field interactions, and identifying the most effective treatment combinations to facilitate the clinical application of PEMF and LIPUS therapies.

## Figures and Tables

**Table 1:** Comparison of key parameters of PEMF and LIPUS therapy.

	Pulsed Electromagnetic Field (PEMF)	Low-Intensity Pulsed Ultrasound (LIPUS)
Feature/Parameter	Pulsed Electromagnetic Field (PEMF)	Low-Intensity Pulsed Ultrasound (LIPUS)
Mechanism of action	Ion mobilization (Na^+^, Ca^2+^), biochemical cascades (cGMP, cAMP), gene activation, growth factor increase, inflammation modulation (adenosine A2A/A3A, JNK MAPK), osteogenesis	Mechanical phenomena (cavitation, microbubbles, microjets, acoustic streaming), activation of signaling pathways (proliferation, differentiation, migration), modulation of inflammation (TNF-α, IL-1β, IL-6), secretion of EVs and miRNAs
Main applications	Fractures, pain, inflammation, edema, osteoarthritis, intracerebral hemorrhage, intervertebral disk degeneration, cancer therapy, sepsis, angiogenesis	Fractures, bone diseases, joints, tendons, cartilage regeneration, ligaments, diabetic wounds, kidney function, heart, adipose tissue, neurological diseases, cell therapy
Recommended parameters	Trapezoidal/sawtooth signal, intensity 1.2–2 mT, frequency 15–75 Hz	Intensity 200–500 mW/cm^2^, daily/every other day application (1–10 min)
FDA approval	Approved for bone non-unions (1979)	Approved for fresh fractures and delayed unions (1994, 2000)

**Table 2:** Comparison of limitations, side effects and contraindications of PEMF and LIPUS.

Therapy	Limitations	Side Effects	Contraindications
PEMF	Lack of long-term data, need for parameter optimization	No cytotoxicity/genotoxicity in in vitro studies	Electronic implants, pregnancy, cancers
LIPUS	Limited effectiveness in extensive damage, incomplete clinical translation	Potential DNA damage with excessive exposure (unconfirmed in vitro)	Pregnancy, bone growth plates, cancers

## Data Availability

No new data were created or analyzed in this study. Data sharing is not applicable to this article.
